# An integrated model for evaluation of maternal health care in China

**DOI:** 10.1371/journal.pone.0245300

**Published:** 2021-01-28

**Authors:** Qianlong Zhao, Junyi Chen, Fulun Li, Aishu Li, Qian Li

**Affiliations:** 1 Child and Adolescent Physical Examination and Health Management Center, Lanzhou University Second Hospital, Lanzhou, China; 2 Department of Clinical Blood Transfusion, Lanzhou University Second Hospital, Lanzhou, China; 3 Department of Child Psychology and Behavior, Lanzhou University Second Hospital, Lanzhou, China; University of Defence in Belgrade, SERBIA

## Abstract

In recent years, in the context of China’s continuous medical and health reforms, the health status of Chinese women and children has been significantly improved through the continuous efforts of staff at all levels of maternal and child health care institutions. Many indicators in maternal health care have improved significantly, but the speed and magnitude of changes have varied. The purpose of this study is to evaluate the dynamic changes in China’s maternal health status from 2004 to 2018, in order to determine whether China’s medical and health reform measures in recent years have improved maternal health. A total of 6 evaluation indicators from the data of China Health Statistics Yearbook 2019 were selected. Then, based on the multi-criteria decision-making (MCDA) methodology, the entropy weighted technique for order preference by similarity to an ideal solution (TOPSIS), entropy weighted rank-sum ratio (RSR) method and the fuzzy comprehensive evaluation were employed in this study. In addition, sensitivity analysis was engaged to validate the stability and accuracy of the achieved results. The study results shows the ranking values of various methods were not exactly the same, but the overall trend was consistent. Overall, the maternal health care in China improved from 2004 to 2018 year by year, of which the top four were ranked from 2015 to 2018, and relatively poor from 2004 to 2006. This means that the policies and measures implemented in China’s medical and health reform in the past few decades have effectively promoted China’s maternal health care, and this will also provide a theoretical basis for future decisions to promote maternal health care.

## Introduction

Maternal health care is decisive for maternal and newborn health and the health of the entire country [1]. China has the largest group of women and children in the world, any improvement in maternal and child health services or polices will benefit over two-thirds of the total population and beyond [2]. Since the 1790s, almost all the UN assemblies and world summits related to development have placed great emphasis on maternal and child health, and without exception, regraded maternal and child health as the most important indicators to measure the development of a particular country [3]. In 2000, 189 heads of state, including China, signed the Millennium Declaration committing themselves to achieve eight goals for development. The target for Millennium Development Goal 5 was to reduce the maternal mortality ratio by three-quarters between 1990 and 2015 [4].

Previous studies about access and quality of health services between urban and rural residents were limited to examining health insurance and health care in certain areas in China [5]. A growing number of studies addressed gender variation in access to health care services and outcomes [6, 7]. Considering that there exists little empirical evidence on the disparity of different groups of populations by distinguishing their degree of equality and benefit of China’s national health care system, we conduct this unique study to fill this knowledge gap in China.

China’s health care reform was expected to be implemented in 3 sequential phases according to the macro guideline issued in 2009 [8]. The first phase was from 2009 to 2012, which was aimed to push all levels of governments in China tore focus on health development and to lay a foundation for the 2020 goals [8]. As a result, more than 125 billion USD flooded into the health sector from 2009 to 2012, with the following 5 targeted priorities: expanding public health insurance, strengthening primary care, establishing an essential medicine programme, providing public health care services, and conducting pilot reforms of public hospitals [9]. The second phase began in 2012 when the central government issued the 12th 5-year plan for health reform; this phase ended in 2015. Apart from continuing to invest financial resources in the 5 target areas outlined in the 2009–2012 phase, the 2012–2015 phase focused its most attention on public hospital reforms [10]. The reforms of county public hospitals were rolled out county by county. The central government intended to reform its wasteful and inefficient county public hospitals by removing the 15% profit margin for drug sales, changing the price schedule, restructuring the governance structure, changing the payment methods, and improving the management [10]. By the end of 2015, almost all county public hospitals were under the reform. The central government also expedited the reform of hospitals in urban areas [11]. In addition to the 17 pilot cities selected in the 2009–2012 phase, another 83 pilot cities across China were confirmed by the central government. These pilot cities were supposed to be used to determine innovative and feasible approaches for scaling up public hospital reform nationwide by policy experimentation [11]. China’s health reform entered its third phase when the Chinese government announced the 13th 5-year plan for medical reform [12]. In this plan, the central government clearly reconfirmed that it would achieve universal coverage of basic health services for all Chinese citizens by 2020.

Under the above background, the purpose of this study is to evaluate the dynamic changes in the state of maternal health care in China during 2004–2018. A total of 6 evaluation indicators from the data of China Health Statistics Yearbook 2019 were selected. Then, based on the multi-criteria decision-making (MCDA) methodology, the weighted technique for order preference by similarity to an ideal solution (TOPSIS), weighted rank-sum ratio (RSR) method and the fuzzy comprehensive evaluation of maternal health care in China were designed. The significance of this study is to evaluate whether China’s medical and health reform measures in recent years have promoted the maternal health status and to provide a theoretical basis for future maternal and child health decisions.

This paper is organized as follows: Section 1 presents the Introduction. Section 2 provides Literature review. Section 3 describes the method, including the Data sources, the Determination of the Weights, the detailed calculation steps of weighted TOPSIS, weighted RSR method and Fuzzy comprehensive evaluation, and the sensitivity analysis process of MCDM methodology. Section 4 shows the application of the different MCDA methods. Section 5 performs a sensitivity analysis to weights of MCDA. Section 6 presents the discussion of results. Finally, conclusions and the future work direction are given in the Section 7.

## Literature review

MCDA is a set of methods used to solve complex problems with multiple standards and objectives. A large number of previous studies have shown MCDA was widely used in many fields, such as management and engineering [13, 14], economics [15], environment [16], political economy [17], and social system [18]. The application in the medical and health field has been booming since the 2000s [19]. Generally, MCDA research methods can be divided into three categories: value measurement models, ranking models and goal programming models. The common ranking methods of MCDA include simple additive weighting (SAW) method [20], TOPSIS method [21], preference ranking organization method for the enrichment of evaluations (PROMETHEE) method [22], analytic hierarchy process (AHP) method [23], artificial neural networks (ANN) method [24], and RSR method [25].

The TOPSIS method is one of the known classical multi-criteria decision-making methods, Which was first developed by Hwang and Yoon that can be used with both normal numbers and fuzzy numbers [26]. Previous studies have shown that TOPSIS and its modified versions have been widely used in many issues, for example, business [21], management and engineering [27–29], health care [30, 31], safety [32], environment [33], etc. TOPSIS provides a simple and easy-to-understand platform for arranging and decomposing decision-making problems involving many attributes [30]. TOPSIS is not limited by research factors, its logic is reasonable and easy to calculate [31]. However, the use of classic TOPSIS technology for decision-making is not enough to resolve ambiguity, subjectivity and incomplete information [33]. Santos et al. [34] suggest that combining TOPSIS with other MCDM methods may be more effective and more flexible to solve problems. Gorgij et al. [35] used the entropy-TOPSIS method to rank the quality of groundwater resources. Jena et al. [36] integrated ANN-cross-validation and AHP-TOPSIS model to improve earthquake risk assessment. He et al. [37] developed a simulation-aided PROMETHEE-TOPSIS approach for the selection of the most desirable groundwater remediation strategies.

The RSR method is also one of the commonly used classical multi-criteria decision-making methods, Which was proposed by Chinese scholar and former professor Tian Fengdiao in Chinese Academy of Preventive Medicine in 1988 [38]. With the joint support and efforts of the majority of scholars, the RSR method has been widely used in the comprehensive evaluation of multiple indicators in the field of medical health and others [39]. Wu et al. [25] conducted a comprehensive evaluation of the medical service efficiency of TCM hospitals based on the RSR method. Chen et al. [40] analyzed the influence of China’s national standards based on the non-integer rank sum ratio method. Stevic et al. [41] also applied the RSR method in the comprehensive quality evaluation of fly ash.

In previous studies, there were various methods for selecting standard weights in the MCDA method [42]. According to Lu et al. [43], the evaluation of indicator weights through subjective weighting methods such as survey method, Delphi method, AHP, etc., may cause deviation of indicator weights due to subjective factors. Therefore, Chen et al. [44] suggested using entropy to calculate the weight of the standard, because it is an effective method that can make the decision-making process more reliable and accurate, and there are no major modeling difficulties. Objective fixed weight methods such as entropy can effectively eliminate human interference because they are based on inherent information [45].

Previous studies have shown that fuzzy set theory was used to resolve ambiguity and uncertainty in the decision-making process [46]. Therefore, the combination of fuzzy set and trend will be very suitable for solving ambiguous group decision problems [47]. In recent years, Fuzzy TOPSIS has been widely used by researchers [48]. Solangi et al. [49] used Fuzzy-TOPSIS method to study Pakistan’s sustainable energy planning strategy. Rashidi et al. [50] used fuzzy TOPSIS when selecting suppliers in the supply chain. Shen et al. [51] applied the extended intuitionistic fuzzy TOPSIS method based on the new distance metric to credit risk assessment. Through these research results and the development of TOPSIS method, it can be seen that the fuzzy TOPSIS method is more widely used and more reliable than the traditional TOPSIS method.

## Methods

### Data sources

**Fig 1:**
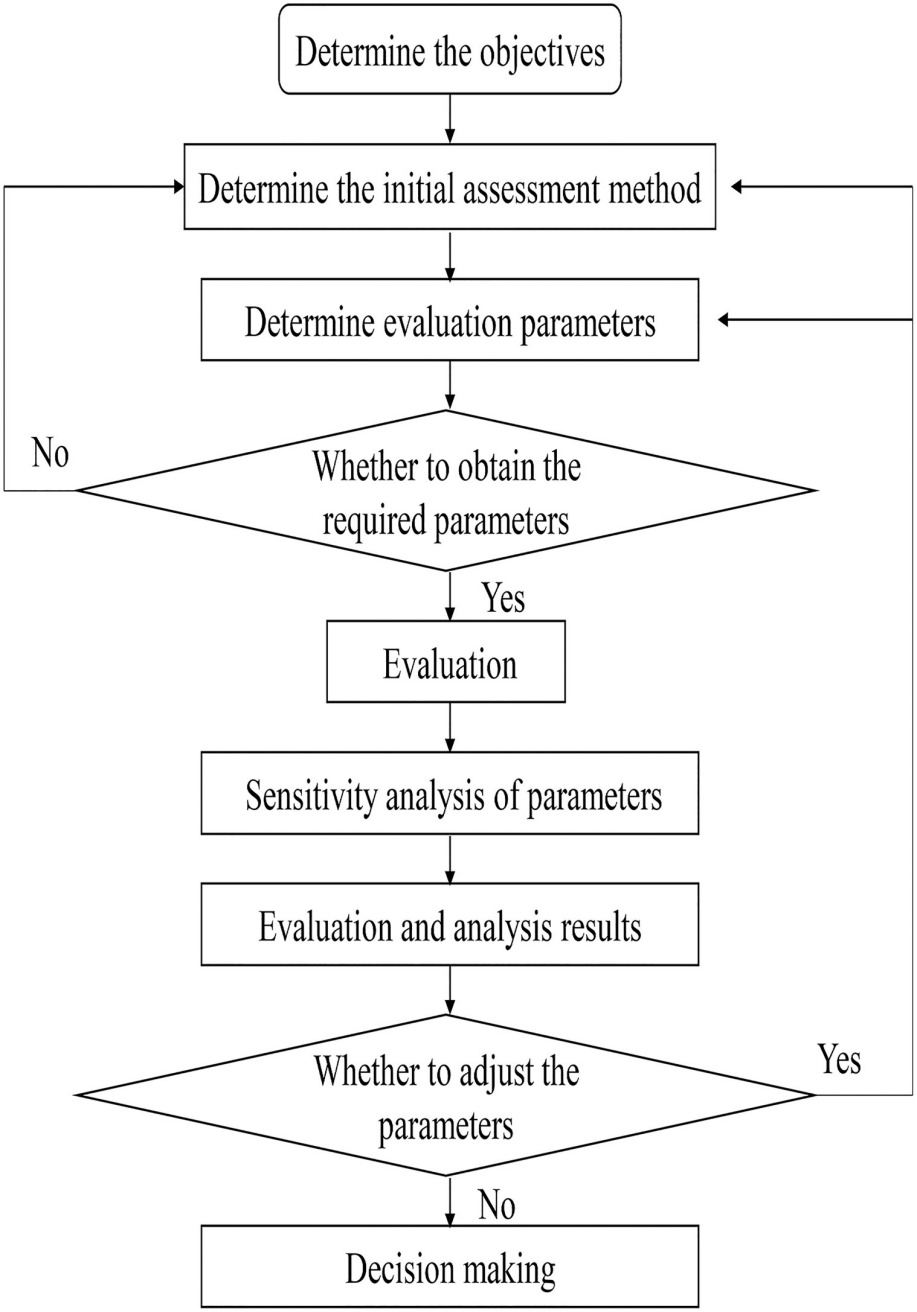
The general model used in this paper.

The research data got from the China Health Statistics Yearbook 2019, which can be get at http://www.cnpub.com.cn/2019/1450795.html?from=singlemessage. According to the relevant data published in the Yearbook, six indicators were selected to comprehensively evaluate and analyze the national maternal health situation. The six indicators *X*_*1*_*~X*_*6*_ are maternal mortality rate (1/100,000), registration rate (%), system management rate (%), prenatal check-up rate (%), postpartum visit rate (%) and hospital delivery rate (%), of which the maternal mortality rate (1/100,000) is the low-quality indicator, and the registration rate (%), system management rate (%), prenatal check-up rate (%), postpartum visit rate (%) and hospital delivery rate (%) are the high-quality indicator. The original data table after co-trending was established (Table 1). Fig 1 shows the general model used in this paper.

**Table 1:** The original data table of maternal health care in China from 2004 to 2018.

Years	*X*_*1*_	*X*_*2*_	*X*_*3*_	*X*_*4*_	*X*_*5*_	*X*_*6*_
**2004**	99951.7	88.3	76.4	89.7	85.9	82.8
**2005**	99952.3	88.5	76.7	89.8	86.0	85.9
**2006**	99958.9	88.2	76.5	89.7	85.7	88.4
**2007**	99963.4	89.3	77.3	90.9	86.7	91.7
**2008**	99965.8	89.3	78.1	91.0	87.0	94.5
**2009**	99968.1	90.9	80.9	92.2	88.7	96.3
**2010**	99970.0	92.9	84.1	94.1	90.8	97.8
**2011**	99973.9	93.8	85.2	93.7	91.0	98.7
**2012**	99975.5	94.8	87.6	95.0	92.6	99.2
**2013**	99976.8	95.7	89.5	95.6	93.5	99.5
**2014**	99978.3	95.8	90.0	96.2	93.9	99.6
**2015**	99979.9	96.4	91.5	96.5	94.5	99.7
**2016**	99980.1	96.6	91.6	96.6	94.6	99.8
**2017**	99980.4	96.6	89.6	96.5	94.0	99.9
**2018**	99981.7	92.5	89.9	96.6	93.8	99.9

### Determination of the weights

Entropy is recommended to calculate the weight of the criteria. The detailed processes are as follows:

1. The indicators were processed in a consistent manner, that is, the directions of the indicators are consistent. Generally, the difference method was used to convert low-quality indicators to high-quality indicators.
2. Formula (1) and (2) are used to normalize high-quality indicators and low-quality indicators, respectively:
  $$a_{ij}=\frac{{\text{X}}_{ij}-\text{min}\{X_{1j},\ldots ,X_{nj}\}}{\text{max}\{X_{1j},\ldots ,X_{nj}\}-\text{min}\{X_{1j},\ldots ,X_{nj}\}}$$ (1)
  $$a_{ij}=\frac{\text{max}\{X_{1j},\ldots ,X_{nj}\}-{\text{X}}_{ij}}{\text{max}\{X_{1j},\ldots ,X_{nj}\}-\text{min}\{X_{1j},\ldots ,X_{nj}\}}$$ (2)
3. Formula (3) is used to calculate the proportion of the *i*th sample under the *j*th index in the index (*p*_*ij*_):
  $$p_{ij}=\frac{a_{ij}}{\sum_{i=1}^{n}a_{ij}}$$ (3)
4. Formula (4) is used to calculate the entropy value of the *j*th index (*e*_*j*_):
  $$e_{j}=-\text{k}\sum_{i=1}^{n}p_{ij}\text{ln}(p_{ij})$$ (4)
  Where $k=\frac{1}{\text{ln}(n)}$. In this study, *n* = 15, *j* = 1, 2…6.
5. Formula (5) is used to calculate the redundancy of calculating information entropy (*d*_*j*_):
  $$d_{j}=1-e_{j}$$ (5)
6. Formula (6) is used to calculate the weight of each indicator (*W*_*j*_):
  $$W_{j}=\frac{d_{j}}{\sum_{j=1}^{m}d_{j}}$$ (6)

### The weighted TOPSIS method

The weighted TOPSIS method ranks according to the proximity of a limited number of evaluation objects to the idealized target, which is a relatively good evaluation of the existing objects [52]. Weighted TOPSIS method is a ranking method that is close to the ideal solution, and it is a commonly used effective method in MCDA [53]. The detailed processes are as follows:

1. The weighted TOPSIS method requires that all indicators change in the same direction, that is, co-trend.
2. The original data matrix after co-trending was normalized to eliminate the influence of the indicator measurement unit, and a normalized matrix A was established. The index conversion formula is (7). Then multiply each index *a*_*ij*_ of the normalized matrix A by its weight coefficient.
  $$a_{ij}=X_{ij}/\sqrt{\sum_{\text{i}=1}^{\text{n}}{\text{X}}_{\text{ij}}{}^{2}}$$ (7)
  Where *X*_*ij*_ represents the value of the *j*th indicator in the *i* year. In this study, *n* = 15, *j* = 1, 2…6.
3. The optimal solution *A*^+^ and the worst solution *A*^-^ was determined by matrix A.
  The optimal solution *A*^+^ consists of the maximum value in each column of the matrix A: *A*_*j*_^+^ = max (a_1j_, a_2j_…a_15j_), *j* = 1, 2…6. The worst solution A^-^ consists of the minimum value in each column of the matrix A: *A*_*j*_^-^ = min (a_1j_, a_2j_…a_15j_), *j* = 1, 2…6. In this study, *A*^+^ = (0.0417, 0.0534, 0.0610, 0.0515, 0.0521, 0.0218), *A*^-^ = (0.0158, 0.0488, 0.0509, 0.0479, 0.0472, 0.0181).
4. The distance *D*_*i*_^+^ and *D*_*i*_^-^ of each evaluation indicators was calculated by Eqs (8) and (9).
  $$D_{i}{}^{+}=\sqrt{\sum_{\text{j}=1}^{\text{m}}{\left({\text{a}}_{\text{ij}}{}^{+}-{\text{a}}_{\text{ij}}\right)}^{2}}$$ (8)
  $$D_{i}{}^{-}\sqrt{\sum_{\text{j}=1}^{\text{m}}{\left({\text{a}}_{\text{ij}}{}^{-}-{\text{a}}_{\text{ij}}\right)}^{2}}$$ (9)
  Where *D*_*i*_^+^ is the distance of the years to the optimal solution, and *D*_*i*_^-^ is the distance of the years to the worst solution.
5. The proximity level of the years to the optimal solution (*C*_*i*_) was calculated by Eq (10).
  $$C_{i}=\frac{D_{i}{}^{-}}{D_{i}{}^{+}+D_{i}{}^{-}}$$ (10)
  Where the value of *C*_*i*_ is between 0 and 1. When the *C*_*i*_ value is closer to 1, it indicates that the maternal health care status in this year is closer to the optimal level.

### The weighted RSR method

The fundamental theory of the weighted RSR method is that a dimensionless statistical indicator is calculated from an n×m matrix using rank conversion. After this calculation, the distribution of WRSR could be explored using parametric statistical methods. Generally, the WRSR indicator ranges from 0 (worst) to 1 (best) and follows a normal distribution. Additionally, the status (worst/best) of subjects could be evaluated using the WRSR order or a set of ordinal classification [38]. The detailed processes are as follows:

1. Rank the indicators of maternal health care, with the high-quality indicators ranked in ascending order and the low-quality indicators ranked in descending order.
2. The value of RSR was calculated by Eq (11).
  $$RSR=\frac{1}{m*n}\sum_{\text{j}=1}^{\text{m}}{\text{R}}_{\text{ij}}$$ (11)
  Where *R*_*ij*_ is the rank of maternal health care indicators in China from 2004 to 2018, *i* = 1, 2, 3…15, *m* is the index number of maternal health care (*m* = 6), *n* is the evaluation years of China maternal health care (*n* = 15).
3. Formula (12) is used to calculate the *WRSR*:
  $$WRSR=\frac{1}{n}\sum_{\text{j}=1}^{\text{m}}{\text{W}}_{\text{j}}{\text{R}}_{\text{ij}}$$ (12)
  Where *W*_*j*_ is the weight of *j*th indicator.
4. Sort according to the *WRSR* value. A larger *WRSR* value indicates better maternal health.

### Fuzzy comprehensive evaluation method

The detailed processes are as follows:

1. The value of *C*_*i*_ and *WRSR* was calculated through weighted TOPSIS method and weighted RSR method, respectively.
2. The value of *C*_*i*_ and *WRSR* was substituted into the formula
  $$W_{1}C_{i}+W_{2}WRSR,$$ (13)
  where *W*_*1*_: *W*_*2*_ is the weight ratio. According to the FUZZY SET theory [54], the weight ratio *W*_*1*_: *W*_*2*_ are taken as 0.1: 0.9, 0.5: 0.5, 0.9: 0.1, respectively.
3. Sort the numerical values calculated according to formula (13), and select the sorting results with different weight ratios that appear more frequently, which is the comprehensive evaluation result. The larger the value, the better the maternal health care.

### Sensitivity analysis

Assuming that *W*_*k*_ changes to *W*_k_*, *k* = 1, 2, 3,…, n, then *W*_k_* = *γ*_*k*_*W*_*k*_, where *γ*_*k*_ is the initial rate of the change of *W*_*k*_, which is a number greater than zero. Because the sum of the weight of each indicator is equal to 1, when *W*_*k*_ changes, the weights of other indicators will change. Formula (14) is used to express the weights after the change.

$$\begin{array}{ll} W_{1}^{’} & =\frac{W_{\text{1}}}{W_{1}+W_{2}+\ldots W_{k}^{*}+\ldots W_{n}}\\ W_{2}^{’} & =\frac{W_{2}}{W_{1}+W_{2}+\ldots W_{k}^{*}+\ldots W_{n}}\\ W_{k}^{’} & =\frac{W_{k}^{*}}{W_{1}+W_{2}+\ldots W_{k}^{*}+\ldots W_{n}}\\ W_{n}^{’} & =\frac{W_{\text{n}}}{W_{1}+W_{2}+\ldots W_{k}^{*}+\ldots W_{n}} \end{array}$$ (14)

Where *W*_*1*_*’*, *W*_*2*_*’*, *W*_*k*_*’*, and *W*_*n*_*’* are the weights of the first, second, *k*th and *n*th indicators after the change of *W*_*k*_. The variable *β*_*k*_ is defined as the unitary variation rate of the change of *W*_*k*_. Therefore, we can get *γ*_*k*_ by formula (15):

$$\gamma_{k}=\frac{\beta_{{}_{k}}-\beta_{{}_{k}}W_{{}_{k}}}{1-\beta_{{}_{k}}W_{{}_{k}}}$$ (15)

In this study, taking the change of *W*_*1*_ as an example, a total of 14 unit change rate schemes were designed, namely *β*_*1*_ = 0.01, 0.02, 0.05, 0.1, 0.2, 0.5, 1, 1.5, 2, 2.5, 3, 3.5, 4, and 4.5. After designing different unitary variation rate of change schemes, the weights of the indicators under these schemes were recalculated, and the *C*_*i*_ values under these schemes were also obtained.

## Results

### The weights of evaluation indicator

Table 2 shows the weight value of each evaluation indicator. The *X*_*3*_ indicator has the highest weight value with 0.2180, and the *X*_*6*_ indicator has the lowest weight value with 0.0809.

**Table 2:** Weight value of each evaluation indicator.

	*X*_*1*_	*X*_*2*_	*X*_*3*_	*X*_*4*_	*X*_*5*_	*X*_*6*_
**Entropy(*e***_***j***_**)**	0.9285	0.8769	0.8649	0.8801	0.8801	0.9498
**Redundancy(*d***_***j***_**)**	0.0715	0.1231	0.1351	0.1199	0.1199	0.0502
**Weight(*W***_***j***_**)**	0.1154	0.1986	0.2180	0.1935	0.1935	0.0809

### Weighted TOPSIS method evaluation of maternal health care in China from 2004 to 2018

**Fig 2:**
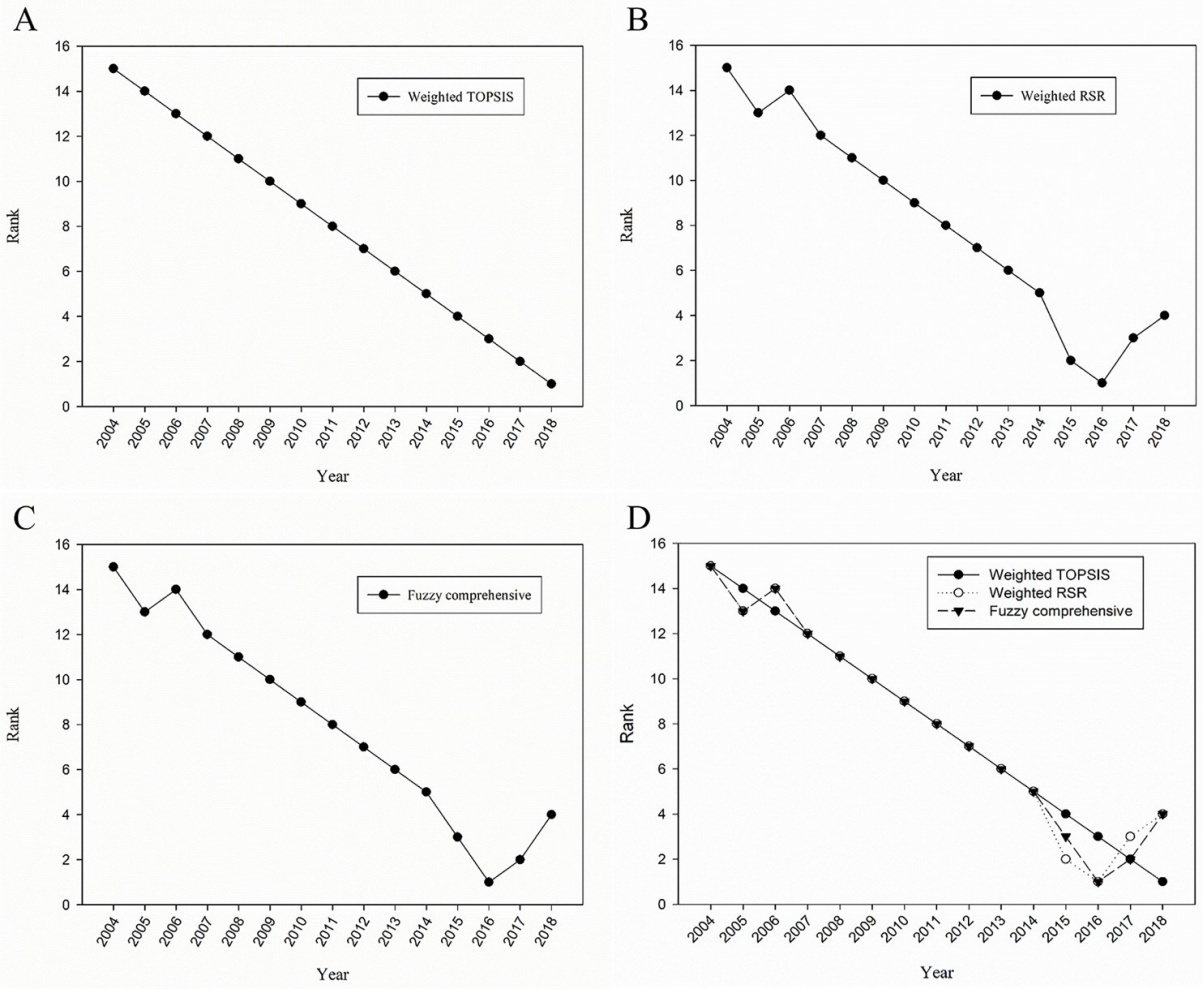
The ranks of alternatives (years) according to different applied methods. A: Weighted TOPSIS; B: Weighted RSR; C: Fuzzy comprehensive; D: Combination Chart of three methods.

According to the *C*_*i*_ value of weighted TOPSIS method evaluation of the maternal health care in each year, the top 4 maternal health care in China from 2004–2018 were in 2018, 2017, 2016 and 2015, and the last four were in 2004, 2005, 2006 and 2007 (Table 3, Fig 2A).

**Table 3:** Weighted TOPSIS method evaluation of maternal health care in China from 2004 to 2018.

Years	*D*_*i*_^+^	*D*_*i*_^-^	*C*_*i*_	Rank
**2004**	0.0291	0.0001	0.0042	15
**2005**	0.0287	0.0029	0.0917	14
**2006**	0.0265	0.0030	0.1025	13
**2007**	0.0239	0.0075	0.2394	12
**2008**	0.0224	0.0092	0.2923	11
**2009**	0.0198	0.0130	0.3955	10
**2010**	0.0173	0.0168	0.4926	9
**2011**	0.0135	0.0197	0.5932	8
**2012**	0.0110	0.0228	0.6741	7
**2013**	0.0090	0.0251	0.7368	6
**2014**	0.0067	0.0271	0.8029	5
**2015**	0.0037	0.0299	0.8888	4
**2016**	0.0034	0.0303	0.9002	3
**2017**	0.0031	0.0300	0.9066	2
**2018**	0.0026	0.0319	0.9253	1

### Weighted RSR method evaluation of maternal health care in China from 2004 to 2018

According to the *WRSR* value ranking evaluation of the maternal health care in each year, the top 4 maternal health care in China from 2004–2018 were in 2016, 2015, 2017, 2018, and the last four were in 2004, 2006, 2005 and 2007 (Table 4, Fig 2B).

**Table 4:** Weighted RSR method evaluation of maternal health care in China from 2004 to 2018.

Years	*X*_*1*_		*X*_*2*_		*X*_*3*_		*X*_*4*_		*X*_*5*_		*X*_*6*_		*WRSR*	Rank
	n	Rank	n	Rank	n	Rank	n	Rank	n	Rank	n	Rank		
**2004**	48.3	1	88.3	2	76.4	1	89.7	1.5	85.9	2	82.8	1	0.0993	15
**2005**	47.7	2	88.5	3	76.7	3	89.8	3	86.0	3	85.9	2	0.1869	13
**2006**	41.1	3	88.2	1	76.5	2	89.7	1.5	85.7	1	88.4	3	0.1138	14
**2007**	36.6	4	89.3	4.5	77.3	4	90.9	4	86.7	4	91.7	4	0.2733	12
**2008**	34.2	5	89.3	4.5	78.1	5	91.0	5	87.0	5	94.5	5	0.3267	11
**2009**	31.9	6	90.9	6	80.9	6	92.2	6	88.7	6	96.3	6	0.4000	10
**2010**	30.0	7	92.9	8	84.1	7	94.1	8	90.8	7	97.8	7	0.4928	9
**2011**	26.1	8	93.8	9	85.2	8	93.7	7	91.0	8	98.7	8	0.5336	8
**2012**	24.5	9	94.8	10	87.6	9	95.0	9	92.6	9	99.2	9	0.6132	7
**2013**	23.2	10	95.7	11	89.5	10	95.6	10	93.5	10	99.5	10	0.6798	6
**2014**	21.7	11	95.8	12	90.0	13	96.2	11	93.9	12	99.6	11	0.7885	5
**2015**	20.1	12	96.4	13	91.5	14	96.5	12.5	94.5	14	99.7	12	0.8745	2
**2016**	19.9	13	96.6	14.5	91.6	15	96.6	14.5	94.6	15	99.8	13	0.9607	1
**2017**	19.6	14	96.6	14.5	89.6	11	96.5	12.5	94.0	13	99.9	14.5	0.8667	3
**2018**	18.3	15	92.5	7	89.9	12	96.6	14.5	93.8	11	99.9	14.5	0.7896	4

### Fuzzy comprehensive evaluation of maternal health care in China from 2004 to 2018

According to the fuzzy comprehensive method evaluation of the maternal health care in each year, the top 4 maternal health care in China from 2004 to 2018 were in 2016, 2017, 2015 and 2018, and the last four were in 2004, 2006, 2005 and 2007 (Table 5, Fig 2C).

**Table 5:** The fuzzy comprehensive evaluation of maternal health care in China from 2004 to 2018.

Years	The fuzzy comprehensive evaluation						
	0.1*WRSR*+0.9*C*_*i*_	Rank	0.5*WRSR* +0.5*C*_*i*_	Rank	0.9*WRSR* +0.1*C*_*i*_	Rank	Comprehensive ranking
**2004**	0.0898	15	0.0521	15	0.0144	15	15
**2005**	0.1720	13	0.1124	13	0.0528	14	13
**2006**	0.1128	14	0.1090	14	0.1051	13	14
**2007**	0.2666	12	0.2400	12	0.2134	12	12
**2008**	0.3206	11	0.2961	11	0.2717	11	11
**2009**	0.3990	10	0.3954	10	0.3918	10	10
**2010**	0.4957	9	0.5072	9	0.5188	9	9
**2011**	0.5420	8	0.5756	8	0.6091	8	8
**2012**	0.6228	7	0.6614	7	0.7000	7	7
**2013**	0.6891	6	0.7261	6	0.7631	6	6
**2014**	0.7928	5	0.8102	5	0.8276	5	5
**2015**	0.8778	2	0.8912	3	0.9047	4	3
**2016**	0.9564	1	0.9394	1	0.9224	3	1
**2017**	0.8722	3	0.8940	2	0.9158	2	2
**2018**	0.8037	4	0.8602	4	0.9167	1	4

Fig 2D shows the results of the weighted TOPSIS, weighted RSR method and the fuzzy comprehensive evaluation were not exactly the same, but the overall trend was consistent. Generally, maternal health care in China improved year by year from 2004 to 2018.

### Correlation analysis

**Fig 3:**
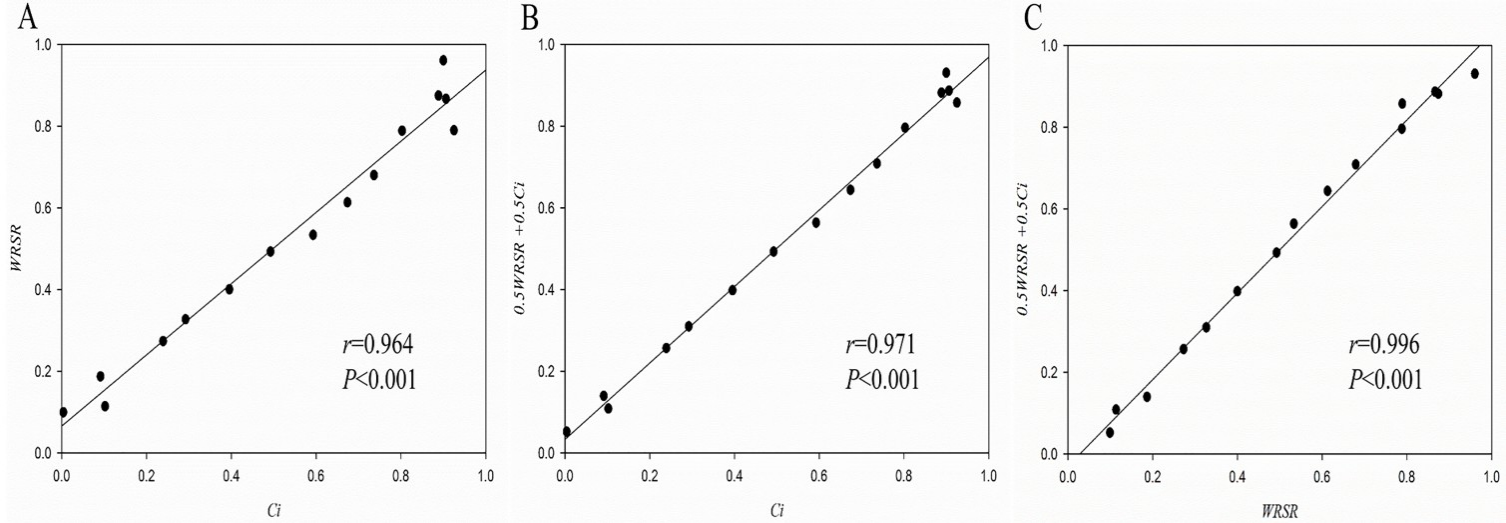
Spearman rank correlation analysis of the results obtained by different methods. A: Correlation between *Ci* and *WRSR*; B: Correlation between *Ci* and 0.5*WRSR* +0.5*C*_*i*_; C: Correlation between *WRSR* and 0.5*WRSR* +0.5*C*_*i*_.

Spearman rank correlation analysis was used to compare the results obtained by different methods (weighted TOPSIS, weighted RSR method, fuzzy comprehensive evaluation). Fig 3A–3C shows the value of *Ci* and *WRSR*, *Ci* and 0.5*WRSR*+0.5*Ci*, *WRSR* and 0.5*WRSR*+0.5*Ci* have a significant positive correlation (*r* = 0.964, *P*<0.001; *r* = 0.971, *P*<0.001; *r* = 0.996, *P*<0.001).

### Sensitivity analysis

**Fig 4:**
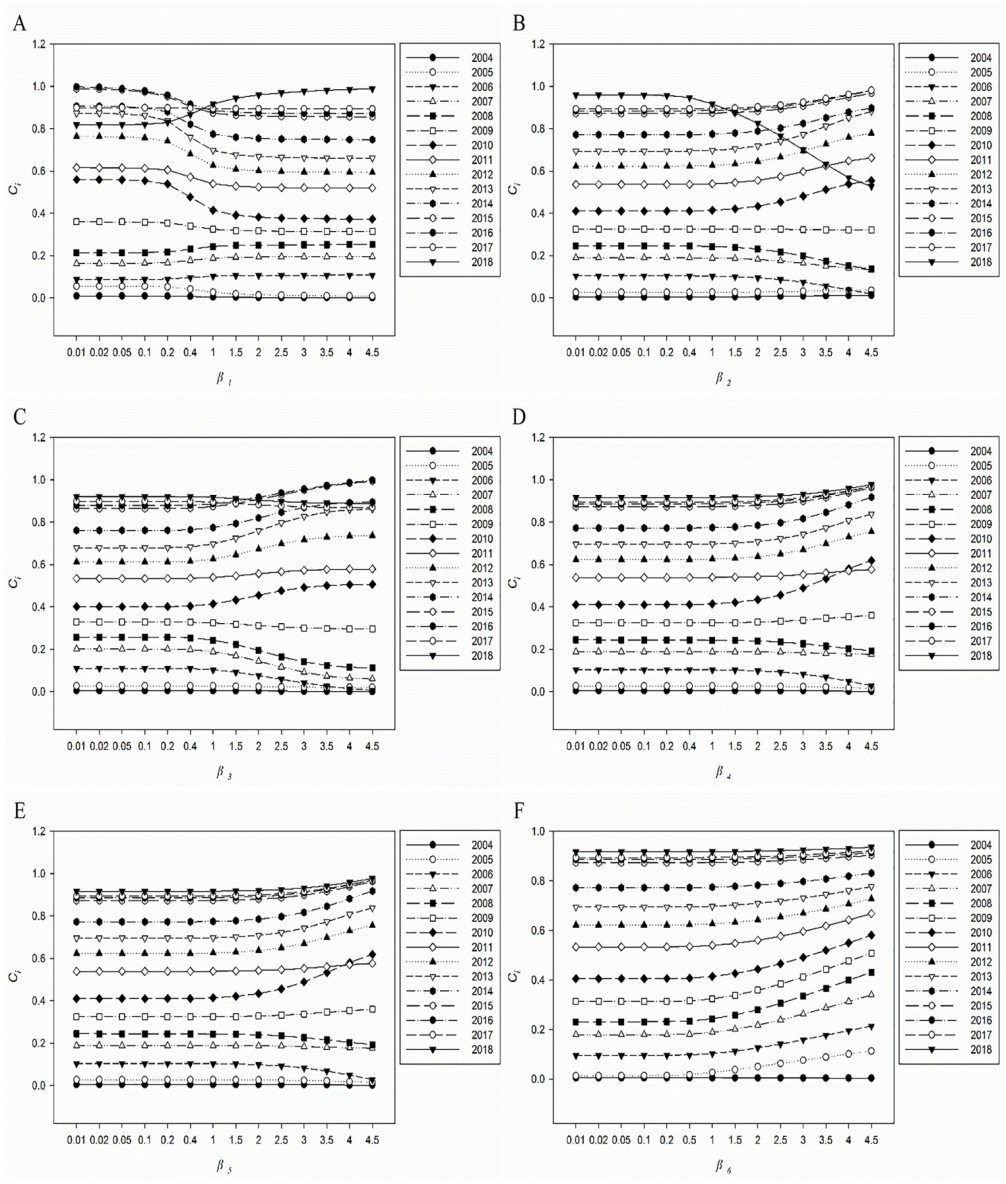
The *C*_*i*_ under different unitary variation ratios for *W*_*k*_. A: *W*_*1*_; B: *W*_*2*_; C: *W*_*3*_; D: *W*_*4*_; E: *W*_*5*_; F: *W*_*6*_.

Table 6 and Fig 4A shows the recalculation results of each weight under different unitary variation ratios for *W*_*1*_. Similarly, the variations of the results under different unitary variation ratios for *W*_*2*_, *W*_*3*_, *W*_*4*_, *W*_*5*_ and *W*_*6*_ can be recalculated, and the results are illustrated in Fig 4B–4F.

**Table 6:** Weights under different unitary variation ratios for *W*_*1*_.

Unitary variation ratio (*β*_*1*_)	Weights					
	*W*_*1*_^*’*^	*W*_*2*_^*’*^	*W*_*3*_^*’*^	*W*_*4*_^*’*^	*W*_*5*_^*’*^	*W*_*6*_^*’*^
**0.01**	0.0012	0.2243	0.2462	0.2185	0.2185	0.0914
**0.02**	0.0023	0.2240	0.2459	0.2183	0.2183	0.0913
**0.05**	0.0058	0.2232	0.2450	0.2175	0.2175	0.0909
**0.1**	0.0115	0.2219	0.2436	0.2162	0.2162	0.0904
**0.2**	0.0231	0.2194	0.2408	0.2137	0.2137	0.0894
**0.5**	0.0462	0.2142	0.2351	0.2087	0.2087	0.0872
**1**	0.1154	0.1986	0.2180	0.1935	0.1935	0.0809
**1.5**	0.1731	0.1857	0.2038	0.1809	0.1809	0.0756
**2**	0.2308	0.1727	0.1896	0.1683	0.1683	0.0704
**2.5**	0.2885	0.1597	0.1754	0.1556	0.1556	0.0651
**3**	0.3462	0.1468	0.1611	0.1430	0.1430	0.0598
**3.5**	0.4039	0.1338	0.1469	0.1304	0.1304	0.0545
**4**	0.4616	0.1209	0.1327	0.1178	0.1178	0.0492
**4.5**	0.5193	0.1079	0.1185	0.1052	0.1052	0.0440

Fig 4A shows the sharp increase of *C*_*i*_ value in 2018 when *β*_*1*_ = 0.2 disturbed the original ranking of 2013–2018. In addition, the ranking of 2015, 2016, and 2017 have changed after *β*_*1*_ = 1. Fig 4B shows the variation of *W*_*2*_ have a great impact on the *C*_*i*_ value in 2018, but other years are not sensitive to variation in *W*_*2*_. Fig 4C shows the ranking of 2015–2018 have changed after *β*_*3*_ = 2. Fig 4D and 4E shows the *C*_*i*_ value in 2010 began to increase from *β*_*4*,*5*_ = 2.5, but the ranking did not exceed 2011 until *β*_*4*,*5*_ = 4. Fig 4F shows the variation of *W*_*6*_ has no effect on the ranking of *Ci* values in each year.

## Discussion

The advantage of the weighted TOPSIS method evaluation is that the method is simple and flexible, and the original data is fully utilized; the disadvantage is that the sensitivity is low and it is sensitive to the interference of outliers [55]. The advantage of weighted RSR method evaluation is that rank is used for calculation, and it is less interference to the outliers; the disadvantage is that some original information may be lost [38]. The advantage of the fuzzy comprehensive evaluation is that it overcomes the shortcomings of the previous two methods, and the analysis of the data is more sufficient and accurate; The final result determined by the principles for choosing more occurrences can accurately reflect the overall change trend [56]. Based on the above analysis, this paper tends to recommend the fuzzy joint comprehensive evaluation method.

In this present study, According to the *C*_*i*_ value ranking of weighted TOPSIS method, the maternal health care in China improved from 2004 to 2018 year by year. According to the WRSR value ranking, in addition to a slight decline in 2005, the maternal health care in China from 2004–2016 improved year by year, and there was a downward trend in 2017–2018 after 2016. According to the fuzzy comprehensive evaluation ranking, the change trend of maternal health care in China from 2004 to 2018 is basically the same as that in weighted RSR. The weight of the standard in this study is calculated using entropy, the weight calculated in this way can effectively eliminate human interference, and the result obtained is more objective. The weight value of each evaluation indicator shows the system management rate indicator (*X*_*3*_) has the highest weight value with 0.2180, and the hospital delivery rate indicator (*X*_*6*_) has the lowest weight value with 0.0809.

Then the sensitivity analysis results of parameter weights shows 2013–2018 are sensitive to variation in *W*_*1*_ and *W*_*3*_, 2010–2018 are sensitive to variation in *W*_*2*_, 2010 and 2011 are sensitive to variation in *W*_*4*_ and *W*_*5*_, none is sensitive to variation in *W*_*6*_. In addition, spearman rank correlation analysis shows the results obtained by multiple different methods are significantly positively correlated. In summary, China’s maternal health care level from 2004 to 2018 has become better and better. This trend of change is inseparable from China’s medical and health reforms in the past few decades. The summary of measures related to the development of maternal health care in China’s medical and health reforms in recent years is as follows:

1990–2008, the maternal health care in China entered the fifth historical development stage. At this stage, with the booming economy, maternal and child health care has received increasingly domestic and international attention [57]. Through the improvement of legislation and investment, China has made great progress in improving maternal and child health. In 1994, the Law of the People’s Republic of China on Maternal and Infant Health Care was issued, which clearly stipulated the policies for maternal and child health care [58]. In 2001, the National Program for Women’s Development in China (2001–2010) was adopted with a main goal of reducing the maternal mortality rate to 39.75 per 100,000 by 2010 [59]. Up to 2008, maternal and child health care hospitals or centers have been established in all provinces, cities, and counties across the country, and even township hospitals have employed maternal and child health care staff [60]. Since the 1990s, the Chinese government has implemented a series of international programs for maternal and child health interventions in cooperation with United Nations International Children’s Fund (UNICEF), United Nations Fund for Population Activities (UNFPA), World Health Organization (WHO), and the World Bank. The Baby-friendly Hospital and Baby-friendly Initiative was the largest international cooperative project between Ministry of Health and UNICEF since China’s reform [61]. In addition, programs such as Strengthening maternal child health (MCH) and family planning (FP) at the Grass-roots Level, Strengthening Essential Health Services in Poor Rural Areas, human immunodeficiency virus (HIV)/ acquired immune deficiency syndrome (AIDS)/ sexually transmitted diseases (STI) Prevention and Control and MCH, Safe Motherhood Initiatives and other international programs have been implemented successfully and have produced a great promotion effect [62].

After 2009, the maternal health care in China entered the sixth historical development stage, the comprehensive health care reform has laid the institutional foundation for the development of maternal and child health care and the promotion of health equity [57]. In 2009, China carried out comprehensive medical reforms in five area, including: (1) providing equal access to basic medical insurance for both urban and rural residents, and increasing the coverage rate to 90% within three years; (2) strengthening the primary medical and health service system; (3) promoting equalization of basis public health services; (4) establishing national essential medicine system; (5) implementing pilot reforms in public hospitals [63]. The reform provides an institutional basis for the development of maternal and child health care, as the maternal and child health care services and interventions have been incorporated into the equalization of public health services. The maternal and child health related services in basic public health services include free medical examinations before pregnancy, systematic health management of pregnant women, subsidies for rural women in hospital delivery, and free treatment of pregnant women with HIV infection [64]. In addition, the ever-increasing capital investment and allocation of health human resources also ensure the continuous development of maternal health care [65]. After the health care reform in 2009, China increased investment in basic public health funds, including maternal and child health care [60]. Most rural areas were providing maternal and child health services, including hospital services, as part of the New Rural Cooperative Medical Scheme (NRCMS) reimbursement [66]. According to the Yearbook 2019, 2009–2018, the average annual growth rate of the number of health technicians, licensed (assistant) doctors and registered nurses in the maternal and child health institutions in China were 7.80%, 5.48% and 12.15%, respectively. The ratio of doctors to nurses in maternal and child health care institutions increased from 1: 0.80 in 2009 to 1: 1.24 in 2018.

## Conclusion

In this present study, the situation of maternal health care in China from 2004–2018 was selected as cases, we have selected 6 indicators from the latest China Health Statistics Yearbook based on previous research reports, and conducted a comprehensive assessment of China’s maternal health care through the weighted TOPSIS method, the weighted RSR method, and the fuzzy combination of the two methods based on the fuzzy set theory, which was used to solve ambiguity and uncertainty in the decision-making process. We also determined the weight of each evaluation index through the entropy weight method, and conducted a sensitivity analysis on the weights of the parameters. Based on the research results, we can conclude that the fuzzy comprehensive evaluation overcome the shortcomings of a single evaluation method, the ranking values of various methods were not exactly the same, but the overall trend was consistent. Overall, the maternal health care in China improved from 2004 to 2018 year by year, of which the top four were ranked from 2015 to 2018, and relatively poor from 2004 to 2006. This means that the policies and measures implemented in China’s medical and health reform in the past few decades have effectively promoted China’s maternal health care, and this will also provide a theoretical basis for future decisions to promote maternal health care.

This study has certain limitations. First of all, the evaluation index system should be representative. However, due to limited access to certain data, some indicators will not be available. Secondly, the research data has a certain lag. For example, this research uses the latest statistical yearbook, but the latest data is released until 2018. Finally, the evaluation results may not be completely consistent due to the different weighting coefficients, but the differences are less. In the future, the integrated model can be applied to more cases to verify the effectiveness of its evaluation. In addition, we will continue to collect the latest yearbook data to track changes in the results after 2018.
